# New library of aminosulfonyl-tagged Hoveyda–Grubbs type complexes: Synthesis, kinetic studies and activity in olefin metathesis transformations

**DOI:** 10.3762/bjoc.6.132

**Published:** 2010-12-06

**Authors:** Etienne Borré, Frederic Caijo, Christophe Crévisy, Marc Mauduit

**Affiliations:** 1 École Nationale Supérieure de Chimie de Rennes, CNRS, UMR 6226, Av. du Général Leclerc, CS 50837 35708 Rennes cedex 7, France; 2 Université Européenne de Bretagne, 35000 Rennes, France; 3 Omega cat system Sàrl - École Nationale Supérieure de Chimie de Rennes, Av. du Général Leclerc, CS 50837 35708 Rennes cedex 7, France

**Keywords:** cross-metathesis, kinetic studies, olefin metathesis, RCM, ruthenium

## Abstract

Seven novel Hoveyda*–*Grubbs precatalysts bearing an aminosulfonyl function are reported. Kinetic studies indicate an activity enhancement compared to Hoveyda’s precatalyst. A selection of these catalysts was investigated with various substrates in ring-closing metathesis of dienes or enynes and cross metathesis. The results demonstrate that these catalysts show a good tolerance to various chemical functions.

## Introduction

**Figure 1:**
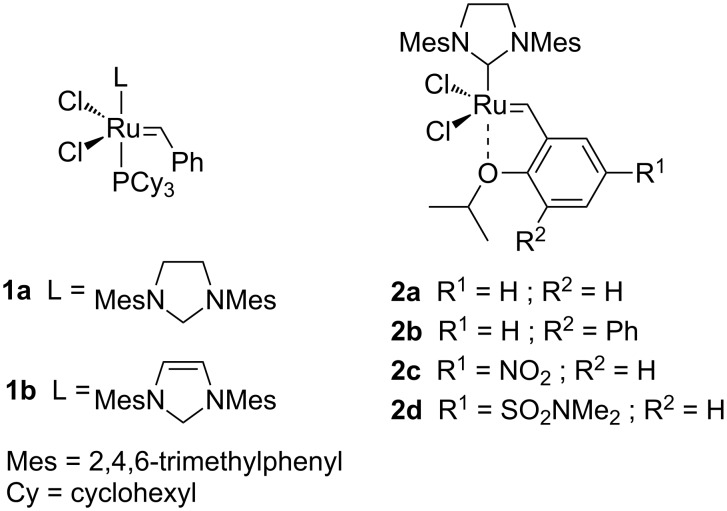
Ruthenium precatalysts for olefin metathesis.

In the last decades, olefin metathesis has become a powerful tool in organic chemistry. Since the discovery of the well-defined ruthenium precatalyst (Cl_2_(PPh_3_)_2_Ru=CHPh) [1], which is tolerant to many functional groups, several synthetic routes (from petrochemical to fine chemical products) have been facilitated [2–4]. However, many research groups have focused their research on the development of more efficient precatalysts (Figure 1). In 1999, Grubbs (**1a**) [5] and Nolan (**1b**) [6] reported ruthenium species bearing one *N*-heterocyclic carbene (NHC) moiety. Despite the stability enhancement of the active species (due to NHC), these catalysts still required a high catalytic loading (up to 20 mol % in some cases [7]). Later, Hoveyda synthesized a recyclable phosphine-free precatalyst (**2a**) [8] based on a release/return concept of the benzylidene ether fragment. Electronic or steric modifications made by Blechert (**2b**) [9–10], Grela (**2c**) [11–12] or Zhan (**2d**) [13] have allowed a decrease of precatalyst loading (down to 1 mol %).

**Figure 2:**
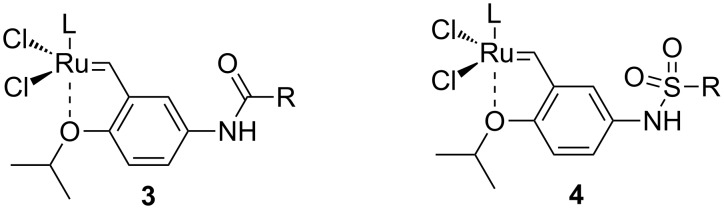
Structure of precatalyst **3** and **4**.

Nevertheless, despite all these recent developments, the problem of the ruthenium contamination in products has still not yet been resolved. Indeed, high concetrations of metal impurities are often present in the final products, limiting industrial applications. Several attempts have been made to reduce the Ru-contamination to <10 ppm, as required by regulatory bodies, for example, by the use of Ru-scavengers, biphasic extraction, silica gel chromatography etc. [14]. Nevertheless, some difficulties remain, for instance: Lower yields are observed when successive silica gel chromatography is performed and some scavengers are very toxic (PbOAc_2_, DMSO…) [15]. Another strategy aims to control the catalyst activity in order to improve recyclability. Recently, various aminocarbonyl-containing “boomerang” precatalysts **3** were synthesized in our laboratory (Figure 2) [16–18]. The results obtained with these catalysts enabled us not only to combine the enhancement of activity with a better stability (1 month in dichloromethane solution) but also to combine it with excellent recyclability (up to 60% at 0.3 mol %). Extremely low levels of Ru-contamination in the final products were determined by ICP-MS analyses (below 6 ppm) after silica gel chromatography. Additionally, a recent study in the synthesis of natural products involving a library of precatalysts **3** [7] shows that the structure of the catalyst must be carefully designed and adapted for a specific transformation.

Owing to this substrate dependency, we focused our attention on the development of a new library of catalysts bearing an aminosulfonyl function **4** (Figure 2).

## Results and Discussion

**Scheme 1:**
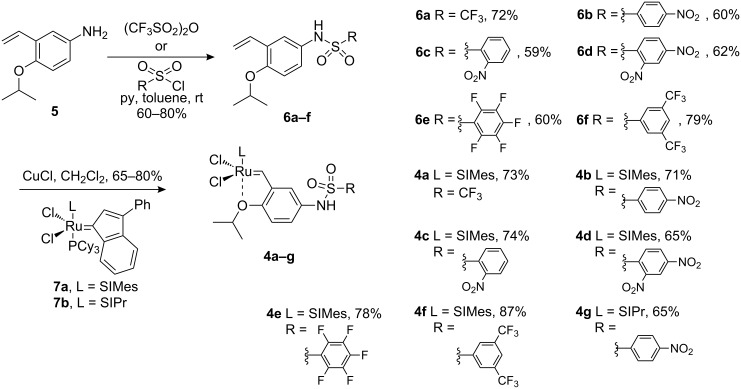
Synthesis of catalysts **4a**–**g**.

To synthesize the catalysts, the required aminosulfonyl function had to be introduced into the styrenylether fragment. The ligands (**6a**–**f**) were synthesized in one step from the previously reported aniline **5** [19–20] and either trifluoromethanesulfonic anhydride or various chlorosulfonyl derivatives (Scheme 1). Ligands **6a**–**f** were isolated in moderate to good yields (60–80%). Their reaction with Ru-indenylidene complex **7a** [21] or **7b** [22] in the presence of CuCl afforded the expected precatalysts **4a**–**g** in good yields.

**Figure 3:**
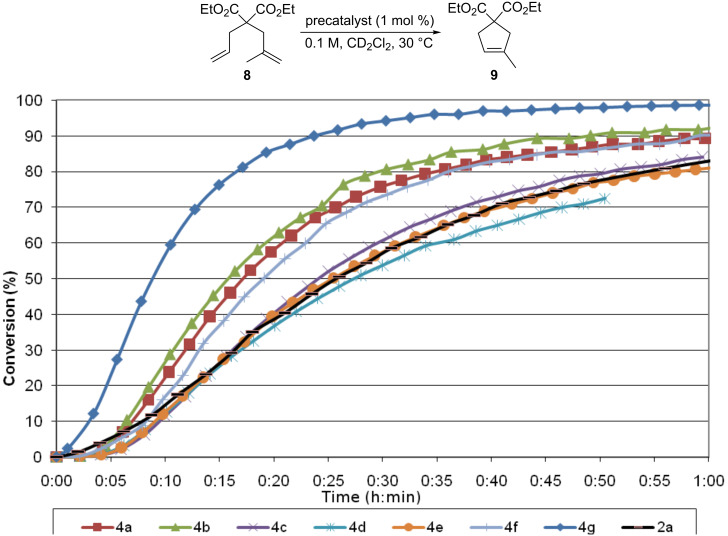
Kinetic studies of RCM of **8** (0.1 M) with precatalysts **4a**–**g** (1 mol %) in CD_2_Cl_2_ at 30 °C.

Then, the reactivity profile of each catalyst was investigated using ^1^H NMR monitored kinetic studies. Comparison between catalysts was done at the initiation step. Moreover, conversions were compared over a reaction time of one hour. The 2-allyl-2-methallylmalonate **8** is usually used as benchmark substrate for ring-closing metathesis, inasmuch it shows significant differences between an activated or a non-activated precatalyst. The reactions were performed at low loading of precatalyst (1 mol %) and 30 °C (Figure 3).

The graphs presented in Figure 3 show three different types of behavior. The catalysts **4c**, **4d** and **4e** are not activated compared to Hoveyda’s complex **2a**. They can be classified as Hoveyda-like complexes. Complexes **4a**, **4b** and **4f** can be considered to be activated catalysts, while catalyst **4g** bearing a more sterically demanding NHC (SIPr) ligand shows a faster initiation compared to its SIMes analogue catalyst **4b**. Additionally, **4g** gave the best conversion over a reaction time of one hour.

In order to investigate potential substrate dependency, the activity of the five SIMes-catalysts **4a**–**e** was evaluated in three different cross-metathesis (CM) reactions involving methyl acrylate, methyl vinyl ketone (MVK) and acrylonitrile as electro-deficient alkenes and the two electron-rich olefins **S1** and **S2** (Table 1).

**Table 1:** Scope of **4a**–**e** for CM transformations^a^.

Entry	Substrates	Product	Time	Catalyst	Conversion (%)*^b,c^*

1 2 3 4 5	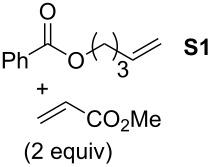	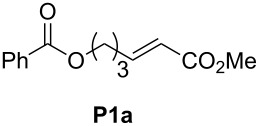	0.5 h	**4a** **4b** **4c** **4d** **4e**	69 50 71 73 62
6 7 8 9 10	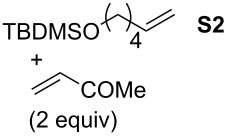	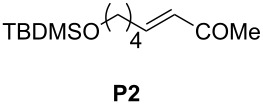	0.5 h	**4a** **4b** **4c** **4d** **4e**	81 88 86 83 80
11 12 13 14 15	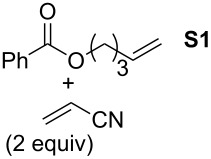	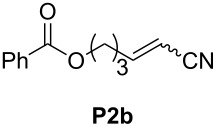	24 h	**4a^d^** **4b^d^** **4c^d^** **4d^d^** **4e^d^**	39 (*E*/*Z* 1/4) 42 (*E*/*Z* 1/4) 24 (*E*/*Z* 1/4) 47 (*E*/*Z* 1/4) 49 (*E*/*Z* 1/4)

^a^Reaction conditions: 1 mol % of catalyst, CH_2_Cl_2_, 0.1 M, rt. ^b^Determined by ^1^H NMR, ^c^*E*/*Z* ratio 20/1, ^d^2 mol % of catalyst, CH_2_Cl_2_, 0.1 M, 40 °C.

In the reaction of methyl acrylate and **S1**, complexes **4c** and **4d** proved to be the most efficient catalysts (entries 1–5) while no clear-cut difference in reactivity was observed when MVK and **S2** were used (entries 6–10). The CM of **S1** and acrylonitrile, which is known to be a demanding substrate, was more problematic since low conversions were observed after 24 h of reaction at 2 mol % catalyst loading (entries 11–15). Unexpectedly, complex **4c** was half as efficient as the other analogues (entry 13). So, the nature of the electron-withdrawing group (EWG) appears to have a rather weak influence on the behaviour of the catalysts in these cases.

Finally, the reactivity profiles of **4a** and **4g** were compared in various metathesis reactions in order to evaluate the influence of the NHC ligand. The last point of our study was the comparison between catalysts in RCM of dienes or enynes and in one CM reaction (Table 2).

**Table 2:** Comparison of **4b** and **4g** in metathesis reactions^a^.

Entry	Substrate	Product	Catalyst	Time	Conversion (%)^b^

1 2	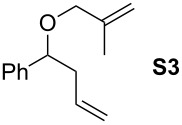	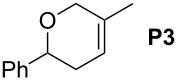	**4b** **4g**	5 h 2 h	62 100
3 4	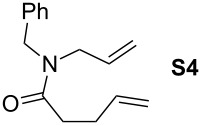	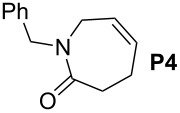	**4b** **4g**	1.75 h 1 h	100 100
5 6	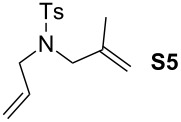	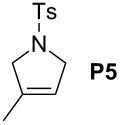	**4b** **4g**	4.5 h 2 h	96 100
7 8	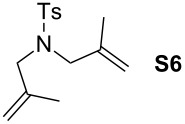	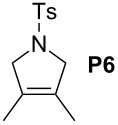	**4b** **4g**	24 h 24 h	18^c^ 3^c^
9 10	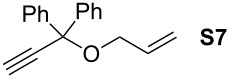	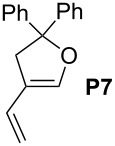	**4b** **4g**	0.75 h 0.5 h	100 100
11 12	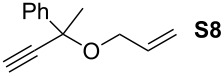	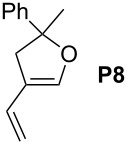	**4b** **4g**	0.5 h 0.5 h	100 100
13 14	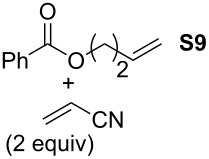	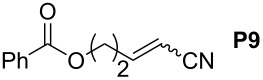	**4b** **4g**	24 h 24 h	48^c^ (*E/Z* 1/4) 84^c^ (*E/Z* 1/4)

^a^Reaction conditions: 1 mol % of catalyst, CH_2_Cl_2_, 0.1 M, rt. ^b^Determined by ^1^H NMR, ^c^2 mol % of catalyst, CH_2_Cl_2_, 0.1 M, 40 °C.

Both catalysts **4b** and **4g** proved to be efficient in all reactions, except in the formation of tetrasubstituted olefin **P6** (entries 7–8). Nevertheless, in almost all cases, either the conversion was higher and/or the reaction duration shorter when SIPr-based complex **4g** was used showing its highest efficiency. This confirms the reactivity profile found in the kinetic study. The outstanding reactivity of **4g** in the CM of **S1** and acrylonitrile must be highlighted since a very good conversion was obtained (entry 14, 84%) [23]. This demonstrates the beneficial combination between the SIPr unit and the electronic activation of the benzylidene fragment.

## Conclusion

A new library of Hoveyda type catalysts bearing aminosulfonyl functions has been synthesized. Their activity profiles have been investigated through kinetic studies and through evaluation of a group of substrates. Most of these have shown high activities, nevertheless the SIPr-based complex **4g** proved to be the most efficient, notably in the case where acrylonitrile was involved in the CM.

## Experimental

Synthesis of 1,1,1-trifluoro-*N*-(4-isopropoxy-3-vinylphenyl)methanesulfonamide (**6a**):To a solution of aniline **5** (40 mg, 0.23 mmol) in dry DCM (3 mL), 2,6-lutidine (54 μL, 0.46 mmol, 2 equiv) was added at 0 °C. Then, trifluoromethanesulfonic anhydride (41 μL, 0.25 mmol, 1.1 equiv) was added dropwise and the mixture was allowed to warm to rt during 12 h. After removal of the solvent under vacuum, the crude product was purified by flash chromatography on silica gel (DCM) to give the expected product as a brown oil (42 mg, 72%). ^1^H NMR (400 MHz, CDCl_3_, δ): 1.35 (d, *J* = 6.1 Hz, 6H, 2 CH_3_), 4.54 (sept, *J* = 6.1 Hz, 1H, CH), 5.30 (dd, *J* = 11.2 Hz and 1.2 Hz, 1H, CH), 5.73 (dd, *J* = 17.7 Hz and 1.2 Hz, 1H, CH), 6.85 (d, *J* = 8.9 Hz, 1H, CH), 6.97 (dd, *J* = 17.7 Hz and 11.2 Hz, 1H, CH), 7.13 (dd, *J* = 8.9 Hz and 2.8 Hz, 1H, CH), 7.36 (d, *J* = 2.8 Hz, 1H, CH). ^13^C NMR (100 MHz, CDCl_3_, δ): 22.0 (2C), 71.2, 114.4, 115.6, 119.8 (q, *J* = 322.8 Hz), 121.4, 125.6, 125.8, 128.9, 130.8, 154.8. ^19^F NMR (376 MHz, CDCl_3_, δ): −74,1 (s, 3F).

Synthesis of *N*-(4-isopropoxy-3-vinylphenyl)-4-nitrobenzenesulfonamide (**6b**): To a solution of aniline **5** (40 mg, 0.23 mmol) in dry toluene (4 mL), were added successively pyridine (37 μL, 0.46 mmol, 2 equiv) and a solution of *p*-nitrobenzenesulfonyl chloride (50 mg, 0.23 mmol, 1 equiv) in 1 mL of toluene. The mixture was stirred at rt overnight. After removal of the solvent under vacuum, the crude product was purified by chromatography (cyclohexane/ethyl acetate, 8:2) to give **6b** as a pale yellow amorphous solid (49 mg, 60%). ^1^H NMR (400 MHz, CDCl_3_, δ): 1.25 (d, *J* = 6.0 Hz, 6H, 2 CH_3_), 4.4 (sept, *J* = 6.0 Hz, 1H, CH), 5.15 (dd, *J* = 11.2 Hz and 1.3 Hz, 1H, CH), 5.52 (dd, *J* = 17.8 Hz and 1.3 Hz, 1H, CH), 6.67 (d, *J* = 8.7 Hz, 1H, CH), 6.83 (dd, *J* = 8.7 Hz and 2.8 Hz, 1H, CH), 6.84 (dd, *J* = 17.8 Hz and 11.2 Hz, 1H, CH), 7.07 (d, *J* = 2,8 Hz, 1H, CH), 7.82 (d, *J* = 9.0 Hz, 2H, CH), 8.19 (d, *J* = 9,0 Hz, 2H, CH). ^13^C NMR (100 MHz, CDCl_3_, δ): 22.0 (2C), 71.1, 114.5, 115.3, 122.6, 124.1 (2C), 124.5, 127.5, 128.6 (2C), 128.8, 130.9, 144.7, 150.1, 154.0.

Synthesis of *N*-(4-isopropoxy-3-vinylphenyl)-2-nitrobenzenesulfonamide (**6c**): Following the procedure described for **6b** using *o*-nitrobenzenesulfonyl chloride, **6c** was obtained as a pale yellow amorphous solid (46 mg, 57%). ^1^H NMR (400 MHz, CDCl_3_, δ): 1.31 (d, *J =* 6.1 Hz, 6H, 2 CH_3_), 4.47 (sept., *J =* 6.1 Hz, 1H, CH), 5.21 (dd, *J =* 11.2 Hz and 1.3 Hz, 1H, CH), 5.59 (dd, *J =* 17.8 Hz and 1.3 Hz, 1H, CH), 6.75 (d, *J =* 8.8 Hz, 1H, CH), 6.91 (dd, *J =* 17.8 Hz and 11.2 Hz, 1H, CH), 7.01 (dd, *J =* 8.8 Hz and 2.7 Hz, 1H, CH), 7.11 (s, 1H, NH), 7.22 (d, *J =* 2.7 Hz, 1H, CH), 7.56 (td, *J =* 7.7 Hz and 1.3 Hz, 1H, CH), 7.69 (td, *J =* 7.5 Hz and 1.4 Hz, 1H, CH), 7.77 (dd, *J =* 7.8 Hz and 1.4 Hz, 1H, CH), 7.85 (dd, *J =* 7.9 Hz and 1.3 Hz, 1H, CH). ^13^C NMR (100 MHz, CDCl_3_, δ): 22.0 (2C), 71.1, 114.4, 115.2, 122.8, 124.9, 125.1, 127.8, 128.6, 130.9, 131.9, 132.2, 134.4, 133.8, 148.2, 154.0.

*N*-(4-isopropoxy-3-vinylphenyl)-2,4-dinitrobenzenesulfonamide (**6d**): Following the procedure described for **6b** using 2,4-dinitrobenzenesulfonyl chloride, **6d** was obtained as a yellow oil (57 mg, 62%). ^1^H NMR (400 MHz, CDCl_3_, δ): 1.31 (d, *J =* 6.1 Hz, 6H, 2 CH_3_), 4.48 (sept., *J =* 6.1 Hz, 1H, CH), 5.24 (dd, *J =* 11.2 Hz and 1.3 Hz, 1H, CH), 5.63 (dd, *J =* 17.8 Hz and 1.3 Hz, 1H, CH), 6.75 (d, *J =* 8.9 Hz, 1H, CH), 6.91 (dd, *J =* 17.8 Hz and 11.2 Hz, 1H, CH), 6.99 (dd, *J =* 8.9 Hz and 2.7 Hz, 1H, CH), 7.24 (d, *J =* 2.7 Hz, 1H, CH), 8.00 (d, *J =* 8.6 Hz, 1H, CH), 8.37 (dd, *J =* 8.6 Hz and 2.2 Hz, 1H, CH), 8.65 (d, *J =* 2.2 Hz, 1H, CH). ^13^C NMR (100 MHz, CDCl_3_, δ): 22.0 (2C), 71.2, 114.2, 115.6, 116.4, 119.0, 122.5, 127.7, 129.2, 129.9, 131.3, 134.7, 140.5, 144.0, 146.3, 152.1.

*N*-(4-isopropoxy-3-vinylphenyl)-2,3,4,5,6-pentafluorobenzenesulfonamide (**6e**): Following the procedure described for **6b** using 2,3,4,5,6-pentafluorobenzenesulfonyl chloride, **6e** was obtained as a red oil (65 mg, 71%). ^1^H NMR (400 MHz, CDCl_3_, δ): 1.32 (d, *J =* 6.1 Hz, 6H, 2 CH_3_), 4.49 (sept, *J =* 6.1 Hz, 1H, CH), 5.27 (dd, *J =* 11.2 Hz and 1.1 Hz, 1H, CH), 5.67 (dd, *J =* 17.8 Hz and 1.1 Hz, 1H, CH), 6.79 (d, *J =* 8.8 Hz, 1H, CH), 6.93 (dd, *J =* 17.8 Hz and 11.2 Hz, 1H, CH), 7.04 (dd, *J =* 8.8 Hz and 2.7 Hz, 1H, CH), 7.19 (s, 1H, NH), 7.25 (d, *J =* 2.7 Hz, 1H, CH). ^13^C NMR (100 MHz, CDCl_3_, δ): 22.0 (2C), 71.2, 114.7, 115.5, 121.1, 123.1, 126.9, 127.5–131.2 (dm, *J* = 245 Hz), 129.0, 130.8,135.3-138.3 (dm, *J =* 256 Hz), 142.2–145.1 (dm, *J =* 259 Hz), 154.1. ^19^F NMR (376 MHz, CDCl_3_, δ): −158 (2F), −144.7 (1F), −136 (2F).

*N*-(4-isopropoxy-3-vinylphenyl)-3,5-bis(trifluoromethyl)benzenesulfonamide (**6f**): Following the procedure described for **6b** using 3,5-bis(trifluoromethyl)benzenesulfonyl chloride, **6f** was obtained as a brown solid (81 mg, 79%). ^1^H NMR (400 MHz, CDCl_3_, δ): 1.32 (d, *J =* 6.1 Hz, 6H), 4.48 ( sept., *J =* 6.1 Hz, 1H), 5.22 (dd, *J =* 1.2 and 11.2 Hz, 1H), 5.57 (dd, *J =* 1.2 and 17.8 Hz, 1H), 6.78 (d, *J =* 8.84 Hz, 1H), 6.92 (m, 2H), 7.02 (s, 1H), 7.12 (d, *J =* 2.7 Hz, 1H), 8.03 (s, 1H), 8.14 (s, 2H). ^13^C NMR (100 MHz, CDCl_3_, δ): 21.9 (2C), 71.3, 114.8, 115.2, 122.4 (q, 270 Hz, 2C), 123.0, 124.9, 126.3 (q, *J =* 3.7 Hz, 2C), 127.2, 127.6 (2C), 129.1, 130.7, 132.7 (q, 34.0 Hz, 2C), 141.5, 154.3. ^19^F NMR (376 MHz, CDCl_3_, δ): −63.1.

General procedure for catalyst formation: To a solution of catalyst **7** and copper chloride (1.1 equiv) in dry DCM (1 mL for 0.02 mmol of Ru-indenylidene complex) was added a solution of **6a**–**f** (1 equiv) in DCM (1 mL for 0.05 mmol of ligand). The resulting mixture was stirred at 35 °C for 5 h. Volatiles were removed under reduced pressure, acetone was added to the residue, and the solution was filtered trough a pad of Celite. The filtrate was concentrated and purified by chromatography on silica gel (pentane/acetone, 75/25) to yield the expected complexes **4a**–**i**.

(1,3-dimesitylimidazolidin-2-ylidene)(2-isopropoxy-5-(trifluoromethylsulfonamido)benzylidene)ruthenium(II) chloride (**4a**): Following the general procedure using the ligand **6a**, complex **4a** was isolated as a green powder (62 mg, 73%). ^1^H (400 MHz, CDCl_3_, δ): 1.13 (d, *J =* 6.1 Hz, 6H, 2 CH_3_), 2.34 (s, 18H, 6 CH), 4.08 (s, 4H, 2 CH_2_), 4.72 (sept, *J =* 6.1 Hz, 1H, CH), 6.54 (d, *J =* 8.7 Hz, 1H, CH), 6.61 (d, *J =* 2.2 Hz, 1H, CH), 6.98 (s, 4H, CH), 7.08 (dd, *J =* 8.7 and 2.2 Hz, 1H, CH), 16.21 (s, 1H, CH). ^19^F (376 MHz, CDCl_3_, δ): -75.72 (s, 3F)

(1,3-dimesitylimidazolidin-2-ylidene)(2-isopropoxy-5-(4-nitrophenylsulfonamido)benzylidene)ruthenium(II) chloride (**4b**): Following the general procedure using the ligand **6b**, complex **4b** was isolated as a green powder (55 mg, 71%). ^1^H NMR (400 MHz, CDCl_3_, δ): 1.21 (d, *J* = 6.1 Hz, 6H, 2 CH_3_), 2.42 (s, 18H, 6 CH), 4.19 (s, 4H, 2CH_2_), 4.84 (sept, *J* = 6.1 Hz, 1H, CH), 6.57 (d, *J =* 7.3 Hz, 1H, CH), 6.71 (bs, 1H, NH), 6.95 (d, *J =* 7.1 Hz, 1H, CH), 7.08 (s, 4H, 4 CH), 7.33 (s, 1H, CH), 7.91 (d, *J =* 7.1 Hz, 2H, 2 CH), 8.27 (d, *J =* 7.3 Hz, 2H, 2 CH), 16.34 (s, 1H, CH).

(1,3-dimesitylimidazolidin-2-ylidene)(2-isopropoxy-5-(2-nitrophenylsulfonamido)benzylidene)ruthenium(II) chloride (**4c**): Following the general procedure using the ligand **6c**, complex **4c** was isolated as a green powder (78 mg, 74%). ^1^H NMR (400 MHz, CDCl_3_, δ): 1.09 (d, *J =* 6.1 Hz, 6H, 2 CH_3_), 2.30 (s, 18H, 6 CH_3_), 4.07 (s, 4H, 2 CH_2_), 4.71 (sept., *J =* 6.1 Hz, 1H, CH), 6.64 (d, *J =* 8.8 Hz, 1H, CH), 6.66 (d, *J =* 2.6 Hz, 1H, CH), 6.95 (s, 4H, 4 CH), 7.15 (s, 1H, CH), 7.33 (dd, *J =* 8.8 and 2.6 Hz, 1H, CH), 7.51 (dt, *J =* 7.7 and 1.2 Hz, 1H, CH), 7.64 (dt, *J =* 7.8 and 1.4 Hz, 1H, CH), 7.70 (dd, *J =* 7.8 and 1.3 Hz, 1H, CH), 7.79 (dd, *J =* 8.0 and 1.1 Hz, 1H, CH), 16.14 (s, 1H, CH).

(1,3-dimesitylimidazolidin-2-ylidene)(5-(2,4-dinitrophenylsulfonamido)-2-isopropoxybenzylidene)ruthenium(II) chloride (**4d**): Following the general procedure using the ligand **6d**, complex **4d** was isolated as a green powder (55 mg, 65%). ^1^H NMR (400 MHz, CDCl_3_, δ): 1.09 (d, *J =* 6.1 Hz, 6H, 2 CH_3_), 2.31 (s, 18H, 6 CH_3_), 4.07 (s, 4H, 2 CH_2_), 4.70 (sept., *J =* 6.1 Hz, 1H, CH), 6.64 (d, *J =* 8.7 Hz, 1H, CH), 6.73 (d, *J =* 2.4 Hz, 1H, CH), 6.96 (s, 4H, 4 CH), 7.33 (dd, *J =* 8.6 and 2.4 Hz, 1H, CH), 7.98 (d, *J =* 8.6 Hz, 1H, CH), 8.28 (dd, *J =* 8.6 and 2.2 Hz, 1H, CH), 8.56 (d, *J =* 2.2 Hz, 1H, CH), 16.17 (s, 1H, CH).

(1,3-dimesitylimidazolidin-2-ylidene)(2-isopropoxy-5-(perfluorophenylsulfonamido)benzylidene)ruthenium(II) chloride (**4e**): Following the general procedure using the ligand **6e**, complex **4e** was isolated as a green powder (69 mg, 78%). ^1^H NMR (400 MHz, CDCl_3_, δ): 1.09 (d, *J =* 6.1 Hz, 6H, 2 CH_3_), 2.33 (s, 18H, 6 CH_3_), 4.08 (s, 4H, 2 CH_2_), 4.72 (sept., *J =* 6.1 Hz, 1H, CH), 6.61 (d, *J =* 8.8 Hz, 1H, CH), 6.67 (d, *J =* 2.5 Hz, 1H, CH), 6.98 (s, 4H, 4 CH), 7.24 (dd, *J =* 8.8 and 2.5 Hz, 1H, CH), 16.20 (s, 1H, CH). ^19^F NMR (376 MHz, CDCl_3_, δ): −159.1 (2F), −145.6 (1F), −137.2 (2F).

(5-(3,5-bis(trifluoromethyl)phenylsulfonamido)-2-isopropoxybenzylidene)(1,3-dimesitylimidazolidin-2-ylidene)ruthenium(II) chloride (**4f**): Following the general procedure using the ligand **6f**, complex **4f** was isolated as a green powder (96 mg, 87%). ^1^H NMR (400 MHz, CDCl_3_, δ): 1.08 (d, *J =* 6.1 Hz, 6H, 2 CH_3_), 2.31 (s, 18H, 6 CH_3_), 4.06 (s, 4H, 2 CH_2_), 4.69 (sept., *J =* 6.1 Hz, 1H, CH), 6.48 (d, *J =* 2.6 Hz, 1H, CH), 6.61 (d, *J =* 8.7 Hz, 2H, CH), 6.95 (s, 4H, 4 CH), 7.26 (dd, *J =* 8.7 and 2.6 Hz, 1H, CH), 7.47 (t, *J =* 7.8 Hz, 1H, CH), 7.60 (t, *J =* 7.7 Hz, 1H, CH), 7.82 (dd, *J =* 13.2 and 7.8 Hz, 2H, 2 CH), 16.13 (s, 1H, CH). ^19^F NMR (376 MHz, CDCl_3_, δ): −58.1 (3F).

(1,3-bis(2,6-diisopropylphenyl)imidazolidin-2-ylidene)(2-isopropoxy-5-(4-nitrophenylsulfonamido)benzylidene)ruthenium(II) chloride (**4g**): Following the general procedure using the ligand **6g**, complex **4g** was isolated as a green powder (88 mg, 65%). ^1^H NMR (400 MHz, CDCl_3_, δ): 1.11 (bd, *J =* 5.3 Hz, 12H, 4 CH_3_), 1.17 (d, *J =* 6.9 Hz, 12H, 4 CH_3_), 1.19 (d, *J =* 6.1 Hz, 6H, 2 CH_3_), 3.45 (sept., *J =* 6.7 Hz, 4H, CH), 4.11 (s, 4H, CH_2_), 4.78 (sept., *J =* 6.1 Hz, 1H, CH), 6.27 (d, *J =* 8.6 Hz, 1H, CH), 6.50 (m, 2H, 2 CH), 7.15 (bs, 1H, NH), 7.29 (d, *J =* 7.8 Hz,4H, CH), 7.46 (m, 2H, CH), 7.73 (m, 2H, 2 CH), 8.13 (m, 2H, 2 CH), 16.16 (s, 1H, CH).

General Procedure for the kinetic reaction: A NMR tube equipped with a septum was filled with diethylallylmethallyl malonate (**8**) (25 mg, 0.1 mmol) and CD_2_Cl_2_ (900 μL) under an argon atmosphere. The sample was equilibrated at 30 °C in the NMR probe. The sample was locked and shimmed before the catalyst addition (100 μL, 1 μmol, 0.01 M solution of catalyst). The reaction progress was monitored by the periodical acquisition of data over 1 h and the conversions were calculated from the integration of allylic protons signals of substrates and products.

General Procedure for Cross-Metathesis Reactions: A Schlenk tube under an argon atmosphere was filled with the activated substrate (0.1 mmol), the unactivated substrate (0.2 mmol, 2 equiv) and CH_2_Cl_2_ (1 mL). Then, the precatalyst solution (0.01 M, 100 μL, 1 μmol) was added. After the required time, the solvent was removed. The conversion was determined by ^1^H NMR.

General Procedure for RCM Reactions: A Schlenk tube under an argon atmosphere was filled with the olefin substrate (0.1 mmol) and CH_2_Cl_2_ (1 mL). Then, the precatalyst (1 μmol) was added. After the required time, the solvent was removed. The conversion was determined by ^1^H NMR.

## Supporting Information

File 1 The Supporting Information contains the ^1^H NMR spectrum of **P8**, the calculation of the substrate/dimer ratio (Table 2, entry 1) and the calculation of product/substrate/dimer ratio (Table 2, entry 14).
