# Supplementary material for: New library of aminosulfonyl-tagged Hoveyda–Grubbs type complexes: Synthesis, kinetic studies and activity in olefin metathesis transformations
Source: Beilstein J Org Chem. 2010 Dec 6;6:1159–66. doi: 10.3762/bjoc.6.132 (PMC3002431; doi:10.3762/bjoc.6.132)
Supplement: File 1 — The Supporting Information contains the 1H NMR spectrum of P8, the calculation of the substrate/dimer ratio (Table 2, entry 1) and the calculation of product/substrate/dimer ratio (Table 2, entry 14). [file Beilstein_J_Org_Chem-06-1159-s001.pdf]

Supporting Information

for

New library of aminosulfonyl-tagged Hoveyda–Grubbs type complexes: Synthesis, kinetic studies and activity in olefin metathesis transformations

Etienne Borré[1,2], Frederic Caijo[3], Christophe Crévisy*[1,2] and Marc Mauduit*[1,2]

Address: [1]École Nationale Supérieure de Chimie de Rennes, CNRS, UMR 6226, Av. du Général Leclerc, CS 50837 35708 Rennes cedex 7, France, [2]Université Européenne de Bretagne, 35000 Rennes, France and [3]Omega cat system Sàrl - École Nationale Supérieure de Chimie de Rennes, Av. du Général Leclerc, CS 50837 35708 Rennes cedex 7, France

Email: Christophe Crévisy* - christophe.crevisy@ensc-rennes.fr; Marc Mauduit* - marc.mauduit@ensc-rennes.fr

* Corresponding author

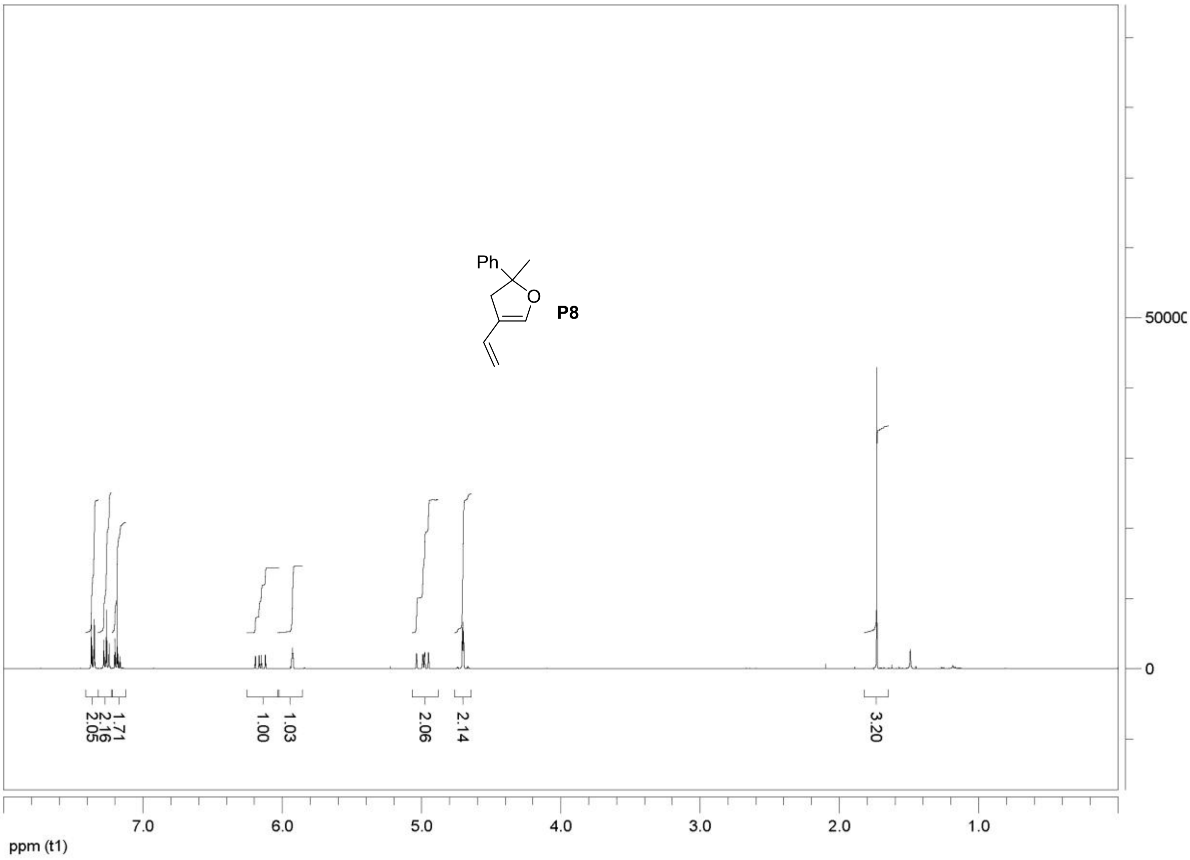

Pure product is obtained in ENYNE metathesis

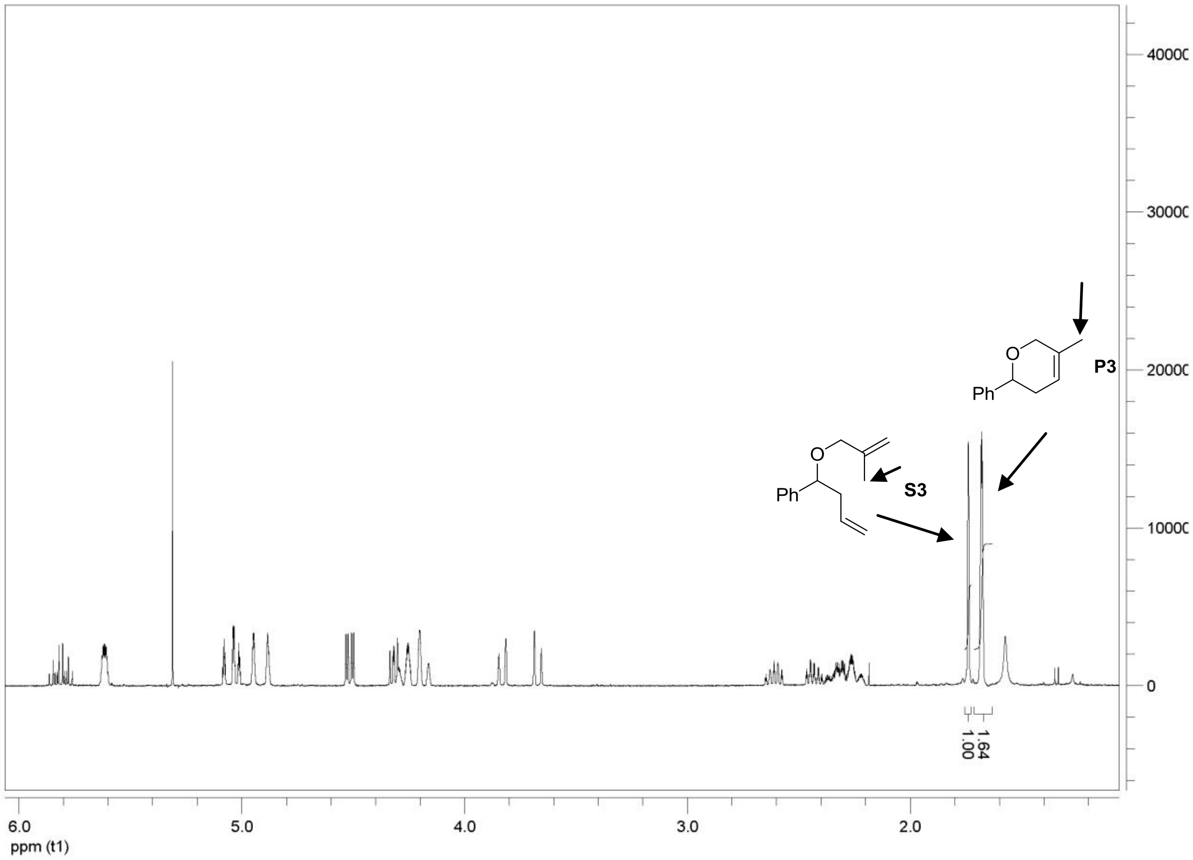

Calculation of substrate/dimer ratio (Table 2, entry 1)

Ratio of product = 100 * 1.64/(1 + 1.64) = 62%

Ratio of substrate = 100 * 1/(1 + 1.64) = 38%

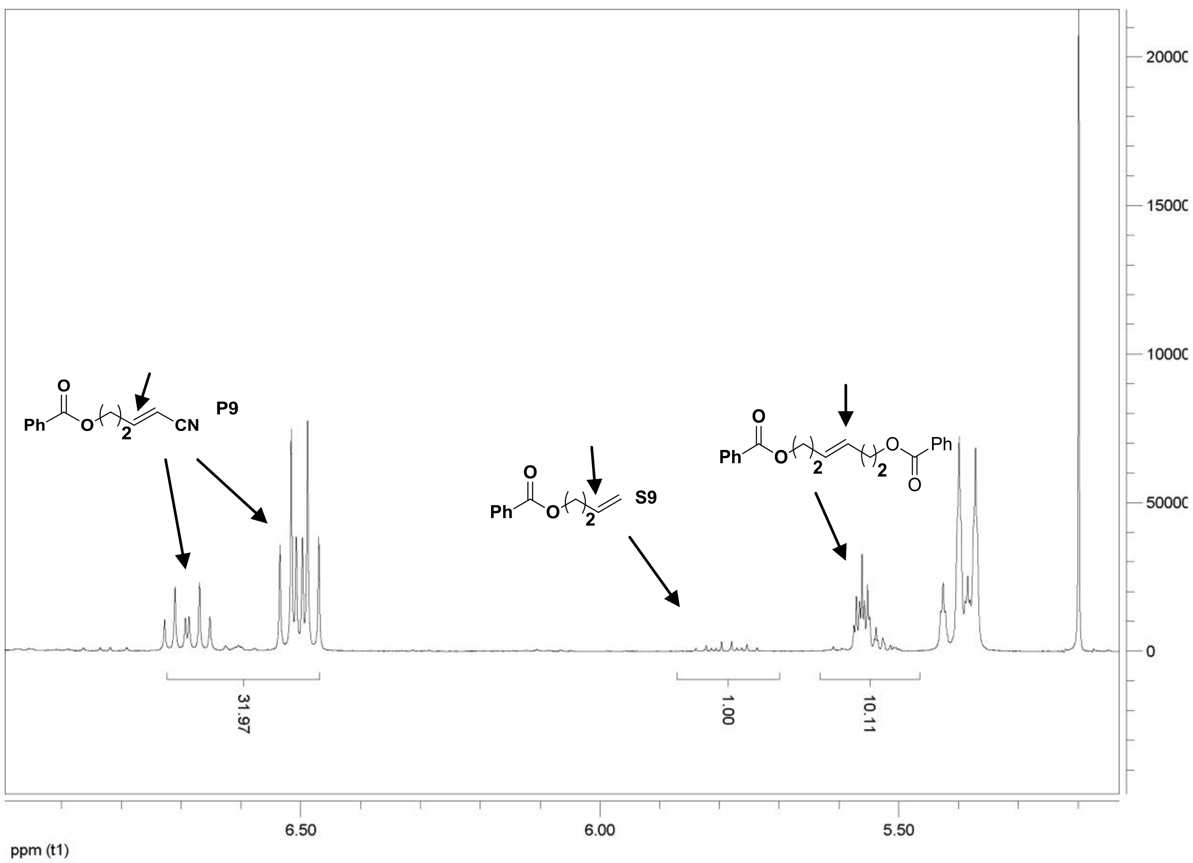

Calculation of product/substrate/dimer ratio (Table 2, entry 14)

Ratio of product = 100 * 31.97/(31.97 + 1 + 10.11/2) = 84%

Ratio of substrate = 100 * 1/(31.97 + 1 + 10.11/2) = 3%

Ratio of dimer = 100 * 10.11/ (2 * (31.97 + 1 + 10.11/2)) = 13%
